# Serum Periostin as a Complementary Biomarker of Frailty in Type 2 Diabetes: A Pilot Study Using the FRAIL Scale

**DOI:** 10.3390/ijms27167503

**Published:** 2026-08-21

**Authors:** Sheila González-Salvatierra, Beatriz García-Fontana, Cristina García-Fontana, Luis Martínez-Heredia, José Francisco Rojas-Pérez, María Carmen Andreo-López, Antonia García-Martín, Manuel Muñoz-Torres

**Affiliations:** 1 Endocrinology and Nutrition Unit, Hospital Universitario Clínico San Cecilio, 18016 Granada, Spain; 2 CIBER of Frailty and Healthy Aging (CIBERFES), Centro de Investigación Biomédica en Red (CIBER), Instituto de Salud Carlos III, 28029 Madrid, Spain; 3 Instituto de Investigación Biomédica de Málaga y Plataforma en Nanomedicina-IBIMA Plataforma BIONAND, 29590 Málaga, Spain; 4 Instituto de Investigación Biosanitaria de Granada (ibs.GRANADA), 18012 Granada, Spain; 5 Department of Cell Biology, University of Granada, 18016 Granada, Spain; 6 Diaverum Málaga, 29002 Málaga, Spain; 7 Department of Medicine, University of Granada, 18016 Granada, Spain

**Keywords:** biomarker, FRAIL scale, frailty, periostin, type 2 diabetes

## Abstract

Frailty is a multidimensional syndrome of reduced physiological reserve that is particularly prevalent in individuals with type 2 diabetes. Identifying objective biochemical markers to complement clinical screening tools, such as the FRAIL scale, is an increasingly important unmet need. Periostin, a matricellular protein involved in tissue remodeling, bone metabolism, and chronic complications of diabetes, has emerged as a potential candidate. In this cross-sectional study of 137 adults with type 2 diabetes (65 ± 8 years), participants were classified as robust, pre-frail, or frail according to FRAIL scores. Frailty correlated positively with age (*p* < 0.001), BMI (*p* = 0.006), waist circumference (*p* = 0.01), and diabetes duration (*p* = 0.040), and negatively with TBS (*p* = 0.001), HDL-c (*p* = 0.040), and eGFR (*p* = 0.009). A stronger positive correlation was observed with serum periostin (*p* < 0.001). Frail individuals showed higher periostin levels than pre-frail (*p* = 0.006) and robust participants (*p* = 0.008), independent of age. Periostin demonstrated moderate discriminatory capacity for frailty status (AUC = 0.710; *p* < 0.001), while adding periostin to clinical variables resulted in a numerical increase in overall model discrimination (AUC = 0.900 vs. 0.858). A threshold of >1307 pmol/L yielded 77.8% sensitivity and 66.7% specificity. These preliminary findings indicate that periostin is significantly associated with frailty in type 2 diabetes and may represent a complementary biochemical biomarker of frailty status, warranting further validation in longitudinal studies.

## 1. Introduction

Frailty is defined as a multidimensional clinical syndrome characterized by decreased physiological and functional reserves, diminished resistance to stressors, and increased vulnerability to adverse outcomes such as falls, hospitalization, and mortality [1]. Although frailty is commonly associated with advanced age [2], emerging evidence shows that it also affects younger populations, including individuals with chronic conditions such as type 2 diabetes [3,4]. The prevalence of frailty in patients with type 2 diabetes is estimated to be three to five times higher than in patients without this disorder [5]. In addition, type 2 diabetes not only increases the risk of frailty but also accelerates its progression [6]. There is evidence that chronic hyperglycemia is associated with the onset of frailty and that glycated hemoglobin levels are associated with its severity [7]. In addition, vascular complications associated with type 2 diabetes are also associated with inactivity and physical and cognitive decline, which may lead to the onset of frailty [8]. This highlights the need for early detection and intervention for frailty in the type 2 diabetes population.

Several instruments have been developed to assess frailty. Among them, the FRAIL scale is one of the most widely used due to its simplicity, ease of administration, and high feasibility across clinical and community settings, while maintaining robust diagnostic accuracy. It comprises five components: Fatigue, Resistance, Ambulation, Illnesses, and Weight Loss [9], and classifies individuals as frail, pre-frail, or robust, thereby facilitating the early identification of individuals at risk of adverse health outcomes [10]. However, despite its clinical utility, there is increasing interest in identifying biochemical markers that provide objective and pathophysiological informed insights into frailty, a need that is particularly relevant in type 2 diabetes, where the prevalence and clinical consequences of frailty are notably high.

Periostin has recently emerged as a potential biomarker of frailty due to its association with physical and cognitive decline in older adults [11]. It is a matricellular protein predominantly expressed in the periosteum and produced by osteoblasts, where it plays an essential role in bone formation, remodeling, and repair [12]. Physiologically, periostin is also involved in the development of teeth, bones, the heart, and the kidneys during embryogenesis [13]. In addition, periostin is involved in tissue repair and regeneration, and elevated levels have been associated with a variety of chronic diseases, including type 2 diabetes [14], bone fragility [15,16], chronic kidney disease [17], and cardiovascular disease [18]. In particular, periostin’s role in extracellular matrix remodeling [13] makes it a promising candidate for the study of frailty, given its impact on organ structure and function.

To date, the association between periostin and frailty in patients with type 2 diabetes has not been studied. Therefore, the aim of this pilot, exploratory study was to investigate whether periostin may serve as a complementary biochemical biomarker of frailty in patients with type 2 diabetes, using the FRAIL scale as the clinical reference for frailty assessment.

## 2. Results

### 2.1. Characteristics of the Study Population According to the FRAIL Scale

Table 1 summarizes the clinical characteristics of the participants classified according to the FRAIL scale as robust (score = 0), pre-frail (score = 1–2), and frail (score = 3–5), while biochemical parameters are presented in Table 2. Significant differences were observed across groups in age, whereas sex distribution remained comparable.

Regarding the clinical evaluation, notable differences emerged in anthropometric measures (BMI and waist circumference), diabetes duration, and the prevalence of dyslipidemia. Additionally, important disparities were detected in macrovascular and microvascular complications, particularly peripheral artery disease and neuropathy, as well as in bone-related outcomes such as fractures and TBS. Post hoc analyses indicated that these differences were mainly observed between the frail group and the robust or pre-frail group (Table 1).

Most biochemical parameters were similar across groups, except for HDL-c and eGFR, which differed significantly across frailty categories. Furthermore, periostin levels were significantly higher in frail individuals compared with the robust and pre-frail groups, supporting its potential relevance as a complementary biochemical biomarker associated with frailty in type 2 diabetes (Table 2).

### 2.2. Determinants of the FRAIL Scale in Patients with Type 2 Diabetes

**Figure 1:**
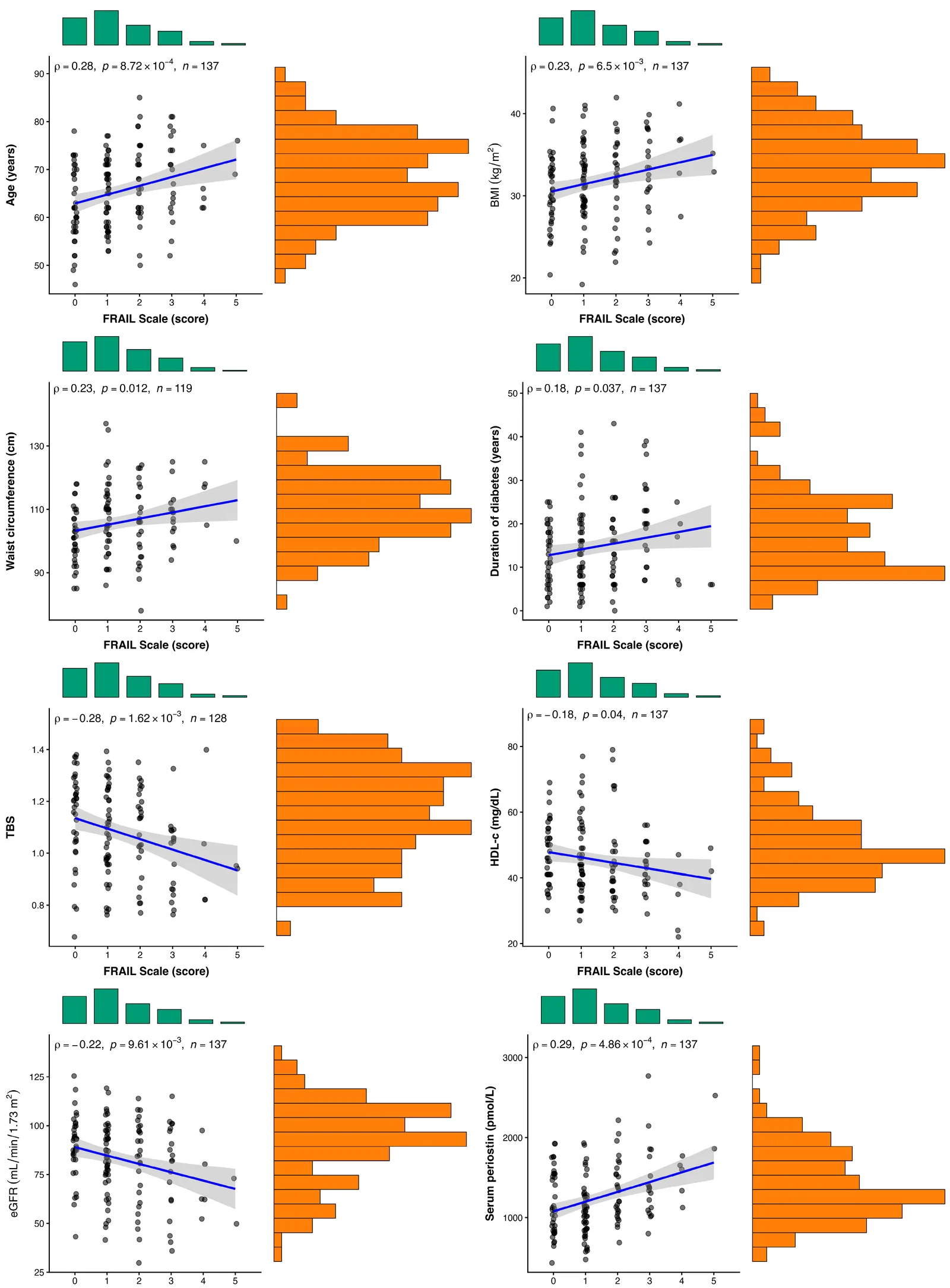
Scatter plots showing the correlation between the Frail scale and age (years), BMI (kg/m^2^), waist circumference (cm), diabetes duration (years), TBS, HDL-c (mg/dL), eGFR (mL/min/1.73 m^2^), and periostin (pmol/L) in patients with type 2 diabetes (*n* = 137). The associations were assessed using Spearman’s correlation coefficients (*p* < 0.05 for each association shown in the scatter plots). BMI: body mass index; TBS: trabecular bone score; HDL-c: high-density lipoprotein cholesterol; eGFR: estimated glomerular filtration rate.

A positive correlation was found between the FRAIL scale scores and factors associated with the development of frailty in patients with type 2 diabetes, such as age (r = 0.28; 95% CI [0.11/0.43]; *p* < 0.001), BMI (r = 0.23; 95% CI [0.06/0.39]; *p* = 0.006), waist circumference (r = 0.23; 95% CI [0.05/0.40]; *p =* 0.01) and duration of diabetes (r = 0.18; 95% CI [0.006/0.34]; *p* = 0.040) (Figure 1). In contrast, we found a negative correlation with TBS (r = −0.28; 95% CI [−0.43/−0.10]; *p* = 0.001), HDL-c (r = −0.18; 95% CI [−0.34/−0.003]; *p* = 0.040) and eGFR (r = −0.22; 95% CI [−0.38/−0.05]; *p* = 0.009) (Figure 1). In addition, we found a stronger positive correlation between the FRAIL scale and the bone marker periostin (r = 0.29; 95% CI [0.13/0.44]; *p* < 0.001) in patients with type 2 diabetes (Figure 1). These findings further support an association between serum periostin levels and frailty status as assessed using the FRAIL scale.

### 2.3. Comparison of Periostin Values According to the FRAIL Scale

**Figure 2:**
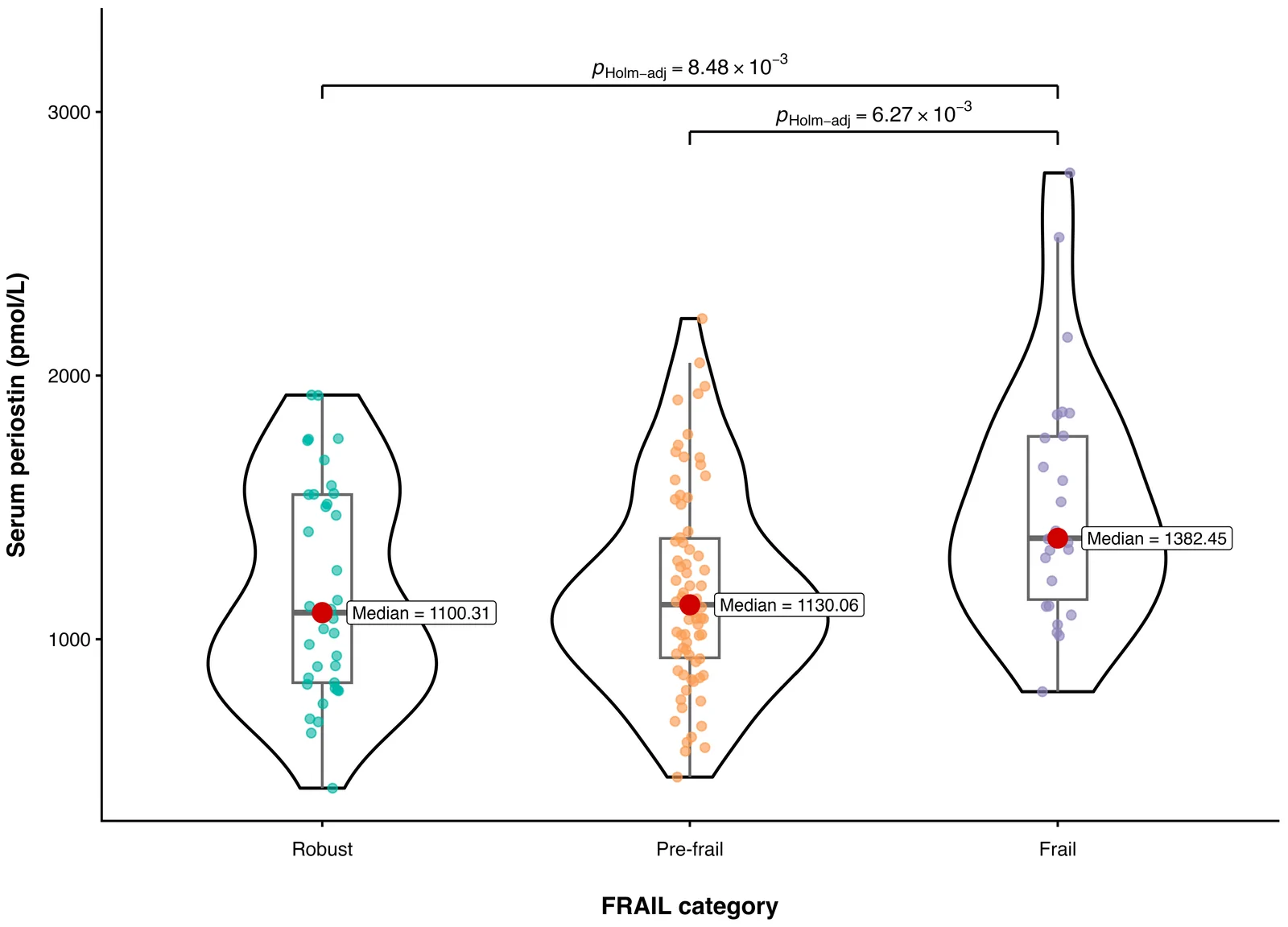
Serum periostin levels in patients with type 2 diabetes according to FRAIL category. Violin plots show the distribution of serum periostin levels in the robust group (score = 0; *n* = 37), pre-frail group (score = 1–2; *n* = 74), and frail group (score = 3–5; *n* = 26). Boxplots represent the median and interquartile range, individual observations are shown as colored points, and the median value is additionally indicated for each group. Between-group differences were assessed using the Kruskal–Wallis test, with Holm-adjusted *p* values shown for statistically significant pairwise comparisons. *p* < 0.05.

No significant differences were observed in serum periostin levels between the control group and the type 2 diabetes group (1209 (972–1452) pmol/L vs. 1147 (939–1536) pmol/L; *p* = 0.966). Furthermore, no significant differences were observed in serum periostin levels between men and women within the type 2 diabetes group (1175 (914–1552) pmol/L vs. 1140 (1007–1408) pmol/L; *p* = 0.780). Similarly, no significant sex differences in serum periostin concentrations were observed within the robust (*p* = 0.354), pre-frail (*p* = 0.961), or frail (*p* = 0.980) categories. However, when patients with type 2 diabetes were categorized according to the FRAIL scale, serum periostin levels were significantly higher in the frail group than in the pre-frail group (1382 (1125–1771) pmol/L vs. 1130 (926–1385) pmol/L; *p* = 0.006), and than in the robust group (1382 (1125–1771) pmol/L vs. 1100 (835–1548) pmol/L; *p* = 0.008). No significant difference was observed between the pre-frail and robust groups (1130 (926–1385) pmol/L vs. 1100 (835–1548) pmol/L; *p* = 0.999) (Figure 2). After adjusting for age, these differences remained significant between the frail and the pre-frail groups (*p* = 0.004), and between the frail and the robust groups (*p* = 0.027).

### 2.4. Determinants of Periostin Levels in Variables Associated with the FRAIL Scale

A multiple linear regression model was used to analyze the variables influencing serum periostin levels. The model included variables correlated with the FRAIL scale: age, BMI, waist circumference, duration of diabetes, TBS, HDL-c, and eGFR. This analysis was performed to explore the potential independent associations between serum periostin levels and variables related to frailty status. The results showed that age was the only variable that was independently associated with serum periostin levels (Β = 15.7; 95% CI [2.64/28.87]; *p* = 0.019).

### 2.5. Effect of Periostin Levels on Frailty in Patients with Type 2 Diabetes According to the FRAIL Scale

Logistic regression modeling was performed to assess the variables related to frailty in patients with type 2 diabetes according to the FRAIL scale. The independent variables included in the model were age, BMI, waist circumference, duration of diabetes, presence of cardiovascular diseases and fractures, TBS, sedentarism, HDL-c, and eGFR, in addition to serum periostin level. Longer diabetes duration (OR = 1.164; 95% CI [1.034/1.307]; *p* = 0.012) and a history of fractures (OR = 15.439; 95% CI [2.167/109.9]; *p* = 0.006) were significantly associated with frailty. Serum periostin was independently associated with frailty (OR = 1.003 per 1 pmol/L increase; 95% CI [1.000/1.005]; *p* = 0.025) in patients with type 2 diabetes. On this scale, a 100 pmol/L higher periostin concentration corresponds to approximately 35% higher odds of frailty. No other variables reached statistical significance in the multivariable model. Sensitivity analyses excluding the seven participants with eGFR < 45 mL/min/1.73 m^2^ yielded materially consistent results. Serum periostin concentrations were 1107 (829–1548) pmol/L in the robust group, 1121 (914–1365) pmol/L in the pre-frail group, and 1386 (1126–1852) pmol/L in the frail group, with the overall between-group difference remaining significant (*p* = 0.004). Serum periostin also remained significantly correlated with FRAIL score (r = 0.28; 95% CI [0.11/0.44]; *p* = 0.001) and was independently associated with frailty in the multivariable model (OR = 1.003; 95% CI [1.000/1.005]; *p* = 0.020). In additional model-specification sensitivity analyses, serum periostin remained significantly associated with frailty after removing fractures, macrovascular complications, and TBS individually and simultaneously from the multivariable model (ORs = 1.002–1.003; all *p* ≤ 0.041).

In sex-specific analyses, higher serum periostin concentrations were significantly associated with higher FRAIL scores in both women (r = 0.353; *p* = 0.006) and men (r = 0.268; *p* = 0.018). In age-adjusted sex-stratified logistic regression analyses, serum periostin was significantly associated with frailty in both women (OR = 1.003 per 1 pmol/L increase; 95% CI [1.001–1.006]; *p* = 0.010) and men (OR = 1.002 per 1 pmol/L increase; 95% CI [1.000–1.003]; *p* = 0.031). No significant sex × periostin interaction was observed (likelihood-ratio χ^2^ = 0.368; *p* = 0.544). Furthermore, after additionally adjusting the primary multivariable model for sex, serum periostin remained independently associated with frailty (OR = 1.004 per 1 pmol/L increase; 95% CI [1.001–1.007]; *p* = 0.021).

**Figure 3:**
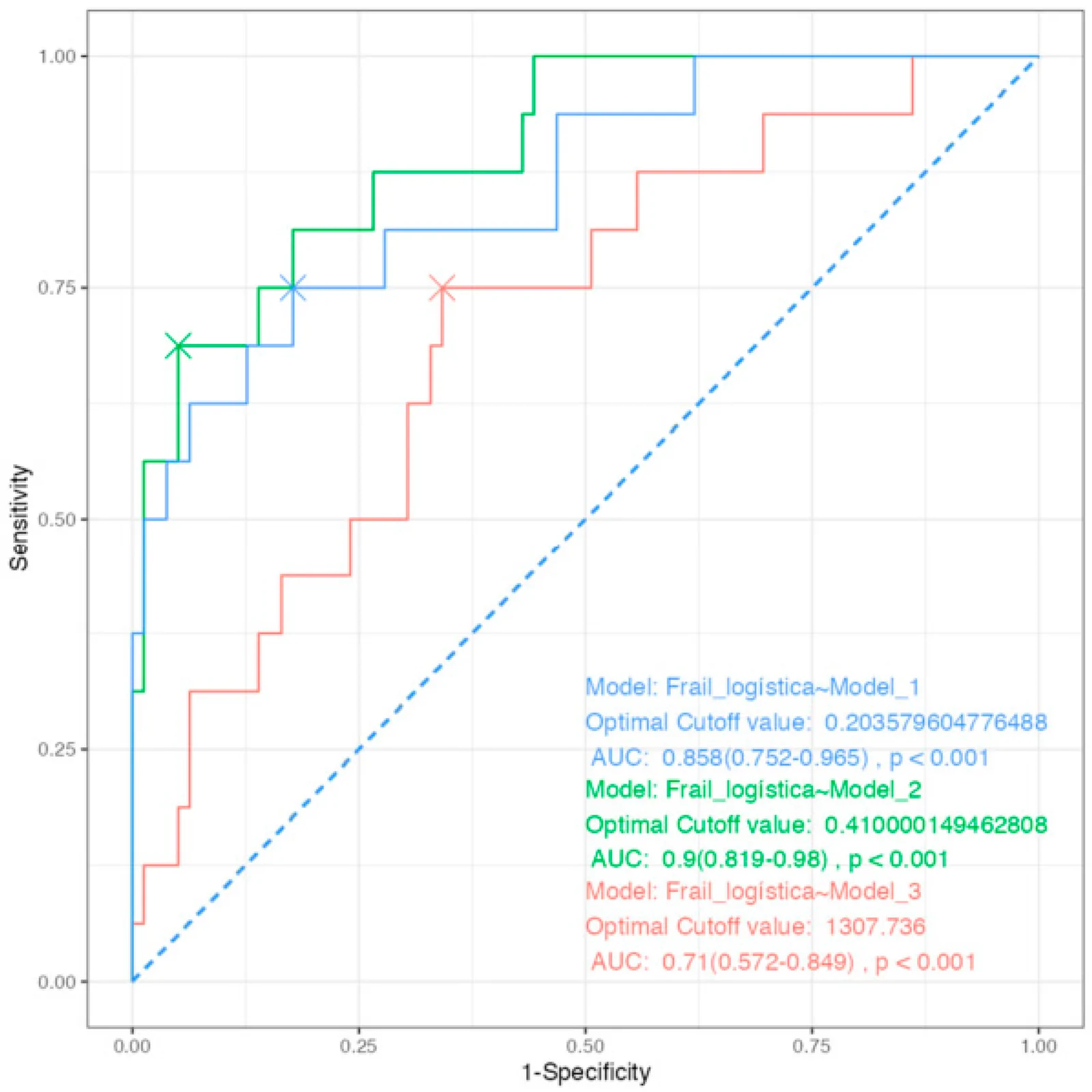
ROC curves comparing the predictive performance of three logistic regression models for frailty. The blue curve corresponds to model_1, which includes all clinical variables associated with the FRAIL scale (age, BMI, waist circumference, duration of diabetes, cardiovascular disease, fractures, TBS, sedentary lifestyle, HDL-c, and eGFR); AUC = 0.858 (*p* < 0.001). The green curve represents model_2, which incorporates the same variables with the addition of serum periostin levels; AUC = 0.900 (*p* < 0.001). The red curve shows model_3, based solely on serum periostin; AUC = 0.710 (*p* < 0.001). Optimal cutoff points derived from Youden’s index are indicated by colored markers on each curve. ROC: receiver operating characteristic; AUC: area under the curve.

To evaluate the discriminatory capacity of serum periostin for distinguishing frail from non-frail individuals, a ROC analysis was performed using three different models. The first model (model_1) included all clinical and metabolic variables associated with the FRAIL scale, namely age, BMI, waist circumference, duration of diabetes, cardiovascular disease, fractures, TBS, sedentary lifestyle, HDL-c, and eGFR. The second model (model_2) incorporated the same set of predictors with the addition of serum periostin levels. A comparison between these two models showed a numerical increase in discriminatory capacity after the inclusion of periostin. Specifically, the AUC of model 1 was 0.858 (95% CI 0.752–0.965; *p* < 0.001), whereas model 2 achieved an AUC of 0.900 (95% CI 0.819–0.980; *p* < 0.001); however, the difference between the two AUCs was not statistically significant (DeLong *p* = 0.213). A third model (model_3), based solely on serum periostin, yielded an AUC of 0.710 (95% CI 0.572–0.849; *p* < 0.001) (Figure 3). Using this model, a periostin threshold of 1307 pmol/L provided a sensitivity of 77.8% and a specificity of 66.7% for detecting frailty in individuals with type 2 diabetes.

## 3. Discussion

Our study is the first to evaluate serum periostin in relation to frailty status in individuals with type 2 diabetes using the FRAIL scale as the clinical reference. The main findings were that serum periostin was positively associated with FRAIL scores, circulating periostin concentrations were higher in patients classified as frail than in those classified as robust or pre-frail, and periostin remained independently associated with frailty in multivariable analyses. Exploratory ROC analyses further suggested a potential incremental contribution of periostin to discrimination when added to clinical variables, although the improvement in discrimination was not statistically significant. Overall, these findings provide preliminary evidence supporting further investigation of serum periostin as a complementary biochemical biomarker associated with frailty in type 2 diabetes.

The FRAIL scale is a simple 5-item screening tool typically used to assess frailty in older adults [10]. The scale demonstrates construct validity through moderate correlations with measures of disability and responsiveness to changes in self-rated health [19] and has been validated in multiple studies, showing good predictive ability for mortality and other adverse outcomes [20]. Frailty is prevalent among patients with type 2 diabetes and is associated with increased risks of adverse health outcomes [21,22]. Chao et al. found that both pre-frailty and frailty, as measured by the FRAIL scale, were associated with increased healthcare utilization and higher risks of mortality, cardiovascular events, hospitalization, and ICU admission in patients with type 2 diabetes [21]. Moreover, longitudinal data from Xu et al. demonstrate that frailty is independently associated with a markedly increased risk of incident type 2 diabetes, likely through shared mechanisms such as inflammation, impaired muscle function, and reduced physical activity [23].

Although frailty is positively associated with advanced age [2], it is not a concern exclusive to the elderly. Evidence shows that frailty also affects middle-aged individuals, posing significant health risks and increasing mortality rates [24,25]. In particular, Hanlon et al. demonstrated that frailty is prevalent among middle-aged adults with type 2 diabetes, with a notable impact on clinical outcomes such as mortality and cardiovascular events [4]. These findings support the concept that frailty in type 2 diabetes is not restricted to very old populations. Regarding our findings on diabetes duration, previous studies have shown that a longer duration of diabetes is associated with an increased risk of frailty. Older adults who have had diabetes for 10 years or more face a higher risk of both frailty and mortality compared to those with a shorter duration of diabetes or normoglycemia [26]. Additionally, elevated HbA1c levels are linked to a higher frailty index and a more rapid decline in frailty status over time [27,28]. Furthermore, our results indicate that frailty is positively correlated with BMI, which is consistent with previous findings showing that a higher BMI is associated with increased frailty [29]. In addition, waist circumference has been shown to be a stronger predictor of frailty than BMI, as it more accurately reflects visceral adiposity and its related metabolic disturbances [30]. Both diabetes and obesity are independently associated with an increased risk of frailty [31]. Yang et al. further demonstrated that the coexistence of diabetes and obesity exacerbates both frailty and kidney dysfunction in patients with chronic kidney disease (CKD) [32]. These findings align with our results, which reveal a negative correlation between frailty and eGFR. In this context, multiple studies have shown that reduced eGFR is associated with a higher risk of frailty in individuals with diabetes. Lee et al. found that community-dwelling older adults with impaired kidney function (eGFR < 30 mL/min/1.73 m^2^) had a significantly increased risk of frailty [33]. Similarly, Wang et al. reported that patients with both diabetes and CKD had a significantly greater risk of developing frailty compared to those without CKD, suggesting that CKD is a strong predictor of frailty regardless of whether it precedes or follows the onset of diabetes [34]. Consistently, Xiong et al. observed an inverse correlation between eGFR and frailty index scores in patients with diabetes [35]. On the other hand, our findings demonstrate a negative correlation between frailty and HDL-c levels. Previous studies have shown that dyslipidemia (particularly altered HDL-c concentrations) is common in individuals with diabetes and may contribute to the development of frailty. Garcia-Esquinas et al. reported that poor glycemic control combined with altered serum lipid profiles, including reduced HDL-c, significantly increases the risk of frailty in diabetic individuals [36]. Reduced HDL2-c and HDL3-c levels have also been associated with impaired glucose tolerance and beta-cell dysfunction in individuals with a family history of type 2 diabetes [37]. In addition to these metabolic and clinical factors, our study also showed a negative correlation between frailty and TBS, indicating that greater frailty is associated with impaired trabecular microarchitecture [38,39]. This is consistent with evidence showing that lower TBS more accurately reflects skeletal deterioration and fracture risk in type 2 diabetes than BMD [40,41,42]. Although all the above parameters are associated with frailty in type 2 diabetes, there is currently no specific analytical biomarker available for its assessment in clinical practice.

Periostin, a matricellular protein, has recently emerged as a potential biomarker of frailty due to its association with age-related functional decline. In this context, Sánchez-Sánchez et al. reported that elevated plasma periostin levels were associated with worsening physical and cognitive performance over a 4-year follow-up period in older adults. Specifically, higher periostin concentrations correlated with decreased gait speed, lower Short Physical Performance Battery (SPPB) scores, and reduced cognitive composite z-scores. These findings support a potential link between periostin and functional decline, a key component of frailty [11]. In contrast, Walsh et al. showed that serum periostin levels are highest during adolescence, when bone modeling is active, but do not differ significantly between middle-aged and older adults, suggesting that the role of periostin in bone health may decrease with age [43]. In murine models, periostin expression has been shown to decline with aging, potentially contributing to impaired fracture healing in older animals. This decrease in periostin is associated with reduced osteoblast activity and bone regeneration capabilities in aged mice compared to younger ones [44]. In our study, age was independently associated with serum periostin levels. Therefore, further research is needed to elucidate the complex relationship between periostin and aging, especially in type 2 diabetes.

Despite growing interest in circulating proteins as potential biomarkers of frailty, a recent proteomic study by Liu et al. identified several plasma proteins associated with frailty in older adults; however, periostin was not among the biomarkers analyzed in their research [45]. To our knowledge, the relationship between serum periostin and frailty has not previously been investigated specifically in individuals with type 2 diabetes.

On the other hand, several studies have demonstrated that periostin is significantly elevated in patients with type 2 diabetes [14], and its presence is closely associated with multiple chronic complications of the disease. Satirapoj et al. reported that urinary periostin levels were elevated in patients with type 2 diabetes and diabetic nephropathy, suggesting its role as a biomarker for renal injury in diabetes [17]. Furthermore, periostin has been associated with diabetic retinopathy, with higher serum levels observed in patients with this complication [46]. In terms of bone metabolism, periostin has also been associated with lower BMD in postmenopausal women with type 2 diabetes, suggesting a role in the development of osteoporosis in this population [16]. More recently, periostin has been implicated in cardiovascular disease associated with type 2 diabetes. Periostin is associated with myocardial fibrosis and heart failure [47] and plays a key role in adverse cardiac remodeling, particularly in diabetic cardiomyopathy. Supporting this, a recent study found that bone-related proteins, including periostin, are associated with increased cardiovascular risk in type 2 diabetes, as measured by the SCORE2-Diabetes algorithm [18]. Renal, cardiovascular, and bone impairment all reduce physiological reserve and functional capacity, which are mechanisms that contribute to frailty. Collectively, these findings indicate that periostin reflects several biological processes relevant to type 2 diabetes and frailty. This pleiotropic profile may limit its specificity when used in isolation, supporting its evaluation as a complementary biomarker within a multidimensional frailty assessment framework.

Finally, we evaluated the discriminatory performance of serum periostin in distinguishing frail from non-frail individuals through ROC analysis. The model based solely on periostin (model_3) demonstrated moderate discriminatory capacity (AUC = 0.710). Notably, incorporating periostin alongside established FRAIL-related clinical variables (model_2) resulted in the highest AUC among the three exploratory models (AUC = 0.900), compared with the clinical model alone (model_1; AUC = 0.858). This numerical increase in discrimination suggests that periostin may provide additional discriminatory information when incorporated into multifactorial assessment models. While the difference between models 1 and 2 did not reach statistical significance, the observed numerical increase in discrimination supports further investigation of periostin as a potential complementary marker for identifying frailty. If validated in independent cohorts, periostin could potentially provide additional biological information alongside existing frailty screening tools, contributing to a more comprehensive assessment of frailty in patients with type 2 diabetes, particularly in settings where extensive functional testing is not feasible. Furthermore, an exploratory, data-derived periostin threshold of 1307 pmol/L was identified as the optimal discriminatory point within this cohort. This threshold requires validation in independent and longitudinal populations before its potential clinical applicability can be established.

From a clinical perspective, several factors should be taken into account when evaluating the potential clinical translation of serum periostin for frailty assessment. Frailty is a complex multisystem condition, and current evidence supports multimarker approaches rather than reliance on a single circulating biomarker, favoring the integration of laboratory biomarkers with established clinical assessments [48,49]. In this context, the moderate discriminatory performance of periostin when used alone in our study supports its potential role as a complementary rather than stand-alone biomarker. Serum periostin can be reliably quantified using immunoassay-based methods [50] and has been investigated as a circulating biomarker in other clinical conditions [51]; however, its routine availability, analytical standardization, and clinically validated thresholds specifically for frailty assessment remain to be established. Moreover, the involvement of periostin in multiple renal, cardiovascular, skeletal, and metabolic processes may limit its disease specificity when interpreted in isolation but may also reflect the multisystem biological burden that characterizes frailty. This is particularly relevant because multimorbidity is incorporated into the FRAIL scale through its Illnesses component. Accordingly, the potential value of periostin may lie in providing biological information on the multisystem processes associated with frailty, while its interpretation should remain integrated within clinical frailty assessment. Finally, the cost-effectiveness of incorporating periostin measurement into frailty assessment has not been established and was not evaluated in the present study. Further prospective studies should therefore determine its reproducibility, incremental clinical utility, and feasibility before routine clinical implementation.

Overall, our findings provide preliminary evidence of an independent association between serum periostin levels and frailty status in patients with type 2 diabetes. While its individual discriminatory performance was moderate when considered in isolation, its consistent association with frailty and the numerical increase in discrimination observed when periostin was incorporated into the multivariable model support its further investigation as a potential complementary biomarker rather than as a replacement for clinical frailty assessment. Given the exploratory nature of this pilot study, the data-derived periostin threshold identified in this cohort should not be considered a clinically validated cutoff. Further investigation in larger, independent and longitudinal cohorts is warranted to validate these findings and determine the potential clinical applicability of periostin within multidimensional frailty assessment strategies.

This study has several limitations that should be acknowledged. First, its cross-sectional design prevents the establishment of causal relationships between periostin levels and frailty. Second, the study population was limited to Spanish Caucasians from a specific geographic region, which may limit the generalizability of our findings to other ethnic or demographic groups. In addition, the concomitant use of common medications such as antihypertensives, lipid-lowering agents, and antidiabetic drugs may have influenced the results and cannot be fully excluded as potential confounders. The relatively small sample size is also a limitation, as it may have reduced the statistical power to detect more subtle associations. In addition, the unequal distribution of participants across FRAIL categories, particularly the smaller number of individuals in the frail group, may have reduced the precision of subgroup estimates and should be considered when interpreting between-group comparisons. Furthermore, the relatively restrictive eligibility criteria inherited from the original cohort, although intended to reduce potential confounding from diseases and treatments affecting bone and metabolic biomarkers, may limit the generalizability of the present findings to the broader population of individuals with type 2 diabetes. Although the association between serum periostin and frailty remained significant after multivariable adjustment and sensitivity analyses, residual confounding cannot be entirely excluded given the complex and multisystem nature of frailty. In particular, unmeasured or incompletely characterized comorbid, nutritional, and lifestyle-related factors may have contributed to residual confounding and should be considered when interpreting the present findings. In addition, the pleiotropic involvement of periostin in multiple biological processes may limit its specificity when interpreted in isolation, and its analytical standardization, clinically validated thresholds, routine implementation, and cost-effectiveness, specifically for frailty assessment, remain to be established. Finally, functional assessments such as muscle strength measurements or performance-based tests were not included, limiting the functional characterization of frailty.

Despite these limitations, this study has notable strengths, including a comprehensive assessment of clinical, anthropometric, and biochemical parameters in a well-characterized patient population and the use of appropriate statistical analyses. To our knowledge, this is the first study to explore the association between serum periostin and frailty status assessed using the FRAIL scale in patients with type 2 diabetes.

## 4. Materials and Methods

### 4.1. Study Population

This pilot, exploratory cross-sectional study included 137 patients with type 2 diabetes (65 ± 8 years, 56.2% male). Type 2 diabetes was diagnosed according to the American Diabetes Association criteria [52]. Patients with type 2 diabetes were consecutively recruited between 2017 and 2018 in the Endocrinology and Nutrition Unit of the University Hospital Clínico San Cecilio of Granada (Spain) according to the following criteria: Caucasian ethnicity and normal blood count values. Participants were excluded if they presented hepatic diseases, defined by aspartate aminotransferase (AST) or alanine aminotransferase (ALT) levels greater than 3 × the Upper Limit of Normal (ULN), or total bilirubin above 1.5 × ULN. Individuals with gastrointestinal diseases, including inflammatory bowel disease (Crohn’s disease or ulcerative colitis) or conditions causing malabsorption, were also excluded. Subjects with active endocrine disorders (except type 2 diabetes) including hyperthyroidism, hypothyroidism, or Cushing’s syndrome, as well as those with bone diseases including osteomalacia, rickets, Paget’s disease, osteogenesis imperfecta, or bone tumors (with the exception of bone fragility), were not eligible for inclusion. Participants receiving thiazolidinediones, warfarin, GLP-1 receptor agonists, or other medications with potential effects on bone metabolism were also excluded. Additionally, individuals with autoimmune diseases, those who had undergone organ transplantation, or who were receiving hormone replacement therapies were excluded from participation. These eligibility criteria largely reflect those established at the time of recruitment of the original cohort and were primarily intended to minimize the potential influence of diseases and treatments that could substantially affect bone metabolism, systemic metabolic status, or circulating bone-related biomarkers. This cohort has been previously described and used in studies evaluating circulating bone-related proteins and their associations with metabolic, renal, and cardiovascular parameters [18,53]. The present study represents a secondary exploratory analysis of this previously established cohort rather than a de novo recruitment specifically designed for frailty assessment.

Additionally, serum samples from 121 healthy blood donors (65 ± 9 years; 56.2% male), matched to the study population by age and sex, were provided by the Biobank of the Andalusian Public Health System (SSPA) and used as a control group for the comparison of serum periostin levels. Healthy controls had no metabolic diseases, including diabetes, or infectious, neoplastic, hepatic, cardiovascular, gastrointestinal, central nervous system, or renal diseases. All control samples were managed by the SSPA Biobank of the University Hospital Clínico San Cecilio (Granada, Spain). Informed consent was obtained from all participants prior to participation. Ethical approval for this study was granted by the Granada Provincial Research Ethics Committee (Project ID: PI15/01207), and the study adhered to the principles outlined in the World Medical Association Declaration of Helsinki [54].

### 4.2. Clinical Evaluation and Biochemical Measurements of the Study Population

Consistent with the cross-sectional design of the study, all clinical, anthropometric, densitometric, and biochemical variables correspond to a single baseline assessment performed at study inclusion. Participants with type 2 diabetes attended scheduled outpatient study assessments and were not hospitalized for study-related procedures. Baseline assessments were performed within the same week. Blood sampling and DXA assessment were performed on the same day when the scheduled DXA appointment was compatible with fasting morning blood collection. When this was not possible, blood sampling was performed on a separate morning, generally within 2–3 days of the DXA assessment. Anthropometric measurements, including height, body weight, and waist circumference, were obtained once during the study visit by trained nurses or dietitians using standardized measurement procedures. Body mass index (BMI) was calculated using the Quetelet formula: weight (kg)/stature (m^2^) [55]. Blood pressure was measured using a validated electronic sphygmomanometer with an appropriately sized cuff, following international recommendations [56]. After a 5 min rest period, two measurements were taken at 1–2 min intervals. When discrepancies greater than 10 mmHg were observed, additional readings were obtained, and the final blood pressure value was calculated as the mean of the last two measurements. Hypertension was defined as systolic blood pressure ≥ 140 mmHg and/or diastolic blood pressure ≥ 90 mmHg, or current use of antihypertensive medication [56]. Dyslipidemia was defined according to established clinical criteria, including low-density lipoprotein cholesterol (LDL-c) > 100 mg/dL, high-density lipoprotein cholesterol (HDL-c) < 50 mg/dL, triglycerides > 150 mg/dL, and/or current treatment with lipid-lowering agents [57]. Macrovascular complications included cerebrovascular disease (transient ischemic attack or ischemic stroke), coronary heart disease (previous myocardial infarction, stable or unstable angina, and coronary revascularization), and ischemic peripheral arterial disease. Microvascular complications included diabetic nephropathy, diabetic retinopathy, and diabetic neuropathy, according to established clinical criteria [57]. Diabetic nephropathy was defined by the presence of microalbuminuria or proteinuria, with albuminuria defined as a urinary albumin-to-creatinine ratio (UACR) ≥ 30 mg/g. Fractures included Colles’ fractures, vertebral fractures, and hip fractures. Bone mineral density (BMD, g/cm^2^) at the lumbar spine (LS), femoral neck (FN), and total hip (TH) was assessed once at baseline in all participants using a Hologic QDR 4500 DXA scanner (Waltham, MA, USA). The coefficients of variation for these measurements were 1.7% for LS, 1.8% for FN, and 1.5% for TH. Corresponding least significant changes (LSCs) were 4.90%, 4.98%, and 4.15%, respectively. Trabecular Bone Score (TBS) at the lumbar spine was obtained with the TBS iNsight software (version 3.0.2.0; Medimaps, Merignac, France). TBS was calculated as the mean of individual values from vertebrae L1–L4 using gray-level texture analysis of the DXA images. The precision error for TBS, expressed as the coefficient of variation, was 1.82%. Osteopenia and osteoporosis were diagnosed by DXA, represented by −2.5 ≤ T-score < −1.0 for osteopenia, and a T-score ≤ −2.5 for osteoporosis. Smoking status, alcohol intake, and physical activity were evaluated using the validated Spanish version of the Rapid Assessment of Physical Activity questionnaire [58].

For biochemical analyses, a single fasting venous blood draw was performed between 08:00 and 10:00 h following an overnight fast in all participants with type 2 diabetes. This restricted morning sampling window was consistently applied throughout the study. A previous study reported modest diurnal variation in serum periostin concentrations, with higher levels observed in the morning than later in the day [59]. Therefore, standardizing blood collection within the same morning interval helped minimize potential variability related to sampling time. Blood was collected into separate tubes according to the intended analysis. Samples required for routine biochemical measurements were processed by the hospital clinical laboratory according to standard clinical procedures. A separate serum collection tube intended for research biomarker analyses, including serum periostin, was gently inverted according to standard collection procedures and allowed to clot for approximately 30 min before transfer to the Biobank of the Andalusian Public Health System (SSPA) for further processing. The sample was subsequently centrifuged at 1500× *g* for 10 min at 4 °C. The separated serum was aliquoted under sterile conditions into 300 μL aliquots and stored at −80 °C until analysis. Fasting plasma glucose (FPG), glycated hemoglobin (HbA1c), lipid profile (LDL-c, HDL-c, total cholesterol, and triglycerides), and serum creatinine were measured using standardized automated laboratory methods. Estimated glomerular filtration rate (eGFR) was calculated using the Chronic Kidney Disease Epidemiology Collaboration (CKD-EPI) equation [60]. Calciotropic hormones and bone-related biomarkers were assessed using commercially available immunoassays. Intact parathyroid hormone (iPTH) concentrations were determined by a two-site immunoassay (Roche Diagnostics GmbH, Mannheim, Germany). Serum 25-hydroxyvitamin D levels were measured by chemiluminescent immunoassay (CLIA) using the Beckman Coulter UniCel DxI 800 platform (Beckman Coulter, Inc., Brea, CA, USA). Alkaline phosphatase (ALP) activity was assessed by a colorimetric method on the AU5800 analyzer (Beckman Coulter, Inc., Brea, CA, USA). Procollagen type I N-terminal propeptide (P1NP) and C-terminal telopeptide of type I collagen (CTX) were quantified by electrochemiluminescence immunoassay (ECLIA; Roche Diagnostics GmbH, Mannheim, Germany). Total osteocalcin was measured using a CLIA method (N-MID Osteocalcin; IDS iSYS automated analyzer; Immunodiagnostic Systems Ltd., Boldon, UK). Serum periostin levels were determined in duplicate in serum aliquots obtained from the same morning fasting blood draw, using an ELISA method according to the manufacturer’s protocol (BI-20433; Biomedica Medizinprodukte GmbH, Vienna, Austria), with a detection range of 20–4000 pmol/L. Precision testing showed intra-assay and inter-assay variations of ≤6% and ≤3% for periostin. According to the manufacturer, the expected mean circulating periostin concentration in healthy controls was 864 pmol/L.

### 4.3. Assessment of Frailty According to the FRAIL Scale

Frailty in all patients was assessed using the FRAIL questionnaire, which evaluates five components: Fatigue, Resistance, Ambulation, Illnesses, and Weight Loss [10]. The FRAIL scale was used according to its original description by Morley et al. [10] and is freely available for academic research use. Fatigue was measured by asking patients how often they felt tired over the past four weeks, with responses of “all of the time” or “most of the time” scoring 1 point. Resistance was evaluated by determining if patients experienced difficulty climbing 10 steps without rest or assistance, while ambulation was assessed by asking if they had difficulty walking several hundred meters without assistance. An affirmative response to either question scored 1 point. For the Illnesses component, a score of 1 was given if patients reported having five or more of the 11 listed chronic illnesses, including hypertension, diabetes, cancer (other than a minor skin cancer), chronic lung disease, heart attack, congestive heart failure, angina, asthma, arthritis, stroke, and kidney disease. Weight loss was scored as 1 for patients who self-reported a weight loss of 5% or more over the past 12 months.

Scores on the FRAIL scale range from 0 to 5, with each component contributing 1 point (0 = best, 5 = worst). Accordingly, patients were classified according to the FRAIL scale as robust (score = 0; *n* = 37), pre-frail (score = 1–2; *n* = 74), and frail (score = 3–5; *n* = 26).

### 4.4. Statistical Analysis

Statistical analyses were performed using SPSS software (version 28.0.1.0; IBM Corp.), RStudio (version 2026.07.1+147; Posit Software, PBC, Boston, MA, USA), and jamovi (version 2.6.26.0). The distribution of continuous variables was assessed using the Kolmogorov–Smirnov test. Continuous variables are presented as mean ± standard deviation (SD) when normally distributed and as median with interquartile range (IQR) when non-normally distributed, while categorical variables are expressed as *n* (%). For comparisons involving normally distributed continuous variables, analysis of variance (ANOVA) was applied, followed by Bonferroni post hoc tests when assumptions of variance homogeneity were met, or Tamhane’s T2 test otherwise. For non-normally distributed variables, between-group comparisons were conducted using the Mann–Whitney *U* test for two groups and the Kruskal–Wallis test for comparisons involving more than two groups. When appropriate, analysis of covariance (ANCOVA) was used to adjust group comparisons for relevant covariates. Categorical variables were compared using the chi-square (χ^2^) test. Associations between continuous variables were evaluated using Spearman’s rank correlation coefficients. Multiple linear regression analysis was performed to identify variables independently associated with serum periostin levels (dependent variable), including variables related to frailty status as assessed by the FRAIL scale as independent predictors. Regression results are reported as unstandardized coefficients (B) with corresponding 95% confidence intervals (CI). To examine whether serum periostin was independently associated with frailty according to the FRAIL scale, multiple logistic regression models were fitted. As a sensitivity analysis, the main analyses involving serum periostin were repeated after excluding participants with eGFR < 45 mL/min/1.73 m^2^. Additional sensitivity analyses were performed by removing fractures, macrovascular complications, and TBS individually and simultaneously from the multivariable model to assess the robustness of the association between serum periostin and frailty. Additional exploratory sex-specific analyses were performed by examining the correlation between serum periostin and FRAIL score separately in women and men, fitting age-adjusted sex-stratified logistic regression models, testing the sex × periostin interaction term, and additionally adjusting the primary multivariable logistic regression model for sex. The discriminative performance of serum periostin for identifying frailty was assessed using receiver operating characteristic (ROC) curve analysis. Predictive accuracy was quantified by the area under the ROC curve (AUC), and AUCs from the clinical models with and without serum periostin were compared using DeLong’s test. Values greater than 0.70 were considered indicative of acceptable discriminatory ability. The optimal cutoff value for periostin was determined using the Youden index. A two-tailed *p* value < 0.05 was considered statistically significant for all analyses.

## 5. Conclusions

This pilot study identified a significant association between serum periostin levels and frailty, as assessed using the FRAIL scale, in individuals with type 2 diabetes. Our findings support further investigation of periostin as a complementary biochemical biomarker associated with frailty in this population. Although its individual discriminatory performance was moderate, the addition of periostin to the multivariable model resulted in a numerical increase in discrimination, suggesting its potential complementary value within a multidimensional frailty assessment framework.

In the context of the current lack of specific analytical biomarkers for frailty in type 2 diabetes, these preliminary findings suggest that periostin may represent a complementary biochemical biomarker within multidimensional frailty assessment strategies. However, validation in larger, independent and longitudinal cohorts is required before its potential clinical utility can be established. Further longitudinal and mechanistic studies are also needed to establish temporal relationships and clarify the biological mechanisms underlying the association between periostin and frailty.

## Figures and Tables

**Table 1:** Clinical characteristics of patients with type 2 diabetes according to the FRAIL scale.

	FRAIL Scale			
	Robust (*n* = 37)	Pre-Frail (*n* = 74)	Frail (*n* = 26)	*p*
Female/Male (*n*)	16/21	30/44	14/12	0.499
Age (years)	62 ± 8	66 ± 7	68 ± 8	**0.007 ^b^**
*Clinical Evaluation*				
Body weight (kg)	84 ± 13	86 ± 15	91 ± 14	0.130
Height (cm)	165 ± 10	164 ± 8	166 ± 9	0.971
BMI (kg/m^2^)	30 ± 4	32 ± 8	34 ± 4	**0.025 ^b^**
Waist circumference (cm)	101 ± 9	107 ± 12	109 ± 9	**0.035 ^a^/0.024 ^b^**
Diabetes duration (years)	13 ± 7	14 ± 9	19 ± 10	**0.016 ^b^/0.048 ^c^**
Systolic blood pressure (mmHg)	134 ± 17	135 ± 18	136 ± 17	0.957
Diastolic blood pressure (mmHg)	81 ± 11	79 ± 9	78 ± 12	0.617
Dyslipidemia, *n* (%)	34 (92)	61 (82)	26 (100)	**0.041**
Hypertension, *n* (%)	31 (84)	62 (84)	24 (92)	0.541
Macrovascular complications, *n* (%)	7 (19)	23 (31)	17 (65)	**<0.001**
Coronary heart disease, *n* (%)	6 (16)	11 (15)	9 (35)	0.077
Cerebrovascular disease, *n* (%)	1 (3)	7 (10)	5 (19)	0.088
Peripheral artery disease, *n* (%)	0 (0)	9 (12)	8 (31)	**0.001**
Microvascular complications, *n* (%)	8 (22)	26 (35)	13 (50)	0.064
Diabetic nephropathy, *n* (%)	5 (14)	12 (16)	7 (27)	0.352
Diabetic retinopathy, *n* (%)	3 (8)	19 (26)	7 (27)	0.074
Diabetic neuropathy, *n* (%)	1 (3)	10 (14)	7 (27)	**0.020**
Fractures, *n* (%)	4 (11)	7 (10)	8 (31)	**0.022**
Osteopenia, *n* (%)	14 (42)	26 (41)	8 (36)	0.896
Osteoporosis, *n* (%)	3 (9)	5 (8)	3 (14)	0.730
LS-BMD (g/cm^2^)	1.03 ± 0.19	1.05 ± 0.2	1.06 ± 0.22	0.829
FN-BMD (g/cm^2^)	0.81 ± 0.16	0.83 ± 0.16	0.81 ± 0.16	0.822
TH-BMD (g/cm^2^)	1.05 ± 0.18	1.05 ± 0.18	1.02 ± 0.17	0.788
TBS	1.14 ± 0.18	1.08 ± 0.17	0.98 ± 0.16	**0.008 ^b^**
Smoker or ex-smoker, *n* (%)	15 (42)	35 (47)	12 (46)	0.855
Alcohol consumption excessive, *n* (%)	7 (19)	14 (19)	1 (4)	0.165
Sedentarism, *n* (%)	3 (11)	7 (11)	8 (36)	**0.014**
*Medication history*				
Insulin, *n* (%)	27 (73)	50 (68)	20 (77)	0.628
Oral antidiabetic drugs, *n* (%)	10 (27)	24 (32)	6 (23)	0.628

BMI: body mass index; BMD: bone mineral density; LS: lumbar spine; FN: femoral neck; TH: total hip; TBS: trabecular bone score. Continuous variables are reported as mean ± standard deviation when normally distributed and as median with interquartile range when distributional assumptions are not met. Categorical variables are presented as *n* (%); percentages were calculated using the available data for each variable. Overall comparisons of continuous variables across FRAIL categories were performed using one-way ANOVA for normally distributed variables and the Kruskal–Wallis test for non-normally distributed variables. When appropriate, pairwise comparisons were performed using Bonferroni-adjusted post hoc tests when homogeneity of variances was met or Tamhane’s T2 test when variances were unequal. Categorical variables were compared using the χ^2^ test. ^a^ Robust vs. Pre-frail; *p* < 0.05; ^b^ Robust vs. Frail; *p* < 0.05; ^c^ Pre-frail vs. Frail; *p* < 0.05. Section headings are shown in italics; significant values in bold.

**Table 2:** Biochemical parameters of patients with type 2 diabetes according to the FRAIL scale.

		FRAIL Scale		
	Robust (*n* = 37)	Pre-Frail (*n* = 74)	Frail (*n* = 26)	*p*
FPG (mg/dL)	144 (123–167)	143 (104–171)	143 (107–202)	0.565
HbA1c (%)	8.0 ± 1.3	7.7 ± 1.4	8.0 ± 1.5	0.384
Total cholesterol (mg/dL)	167 (142–199)	163 (133–186)	153 (121–175)	0.104
HDL-c (mg/dL)	46 (41–54)	44 (38–52)	42 (38–47)	**0.043 ^b^**
LDL-c (mg/dL)	88 (65–118)	89 (61–115)	78 (52–102)	0.158
Triglycerides (mg/dL)	138 (114–191)	134 (95–192)	156 (106–205)	0.999
25-hydroxyvitamin D (ng/mL)	18 ± 6	22 ± 9	20 ± 9	0.054
eGFR (mL/min/1.73 m^2^)	92 (85–99)	86 (71–97)	78 (62–97)	**0.023 ^b^**
ALP (µg/L)	17 (14–22)	16 (13–21)	17 (14–23)	0.935
P1NP (ng/mL)	35 ± 14	37 ± 15	40 ± 15	0.456
CTx (pg/mL)	0.21 (0.11–0.27)	0.19 (0.05–0.31)	0.17 (0.13–0.34)	0.476
iPTH (pg/mL)	45 (35–56)	46 (30–61)	51 (34–65)	0.854
Osteocalcin (ng/mL)	9 (7–13)	8 (7–13)	11 (7–17)	0.445
Periostin (pmol/L)	1100 (835–1548)	1130 (926–1385)	1382 (1125–1771)	**0.008 ^b^/0.006 ^c^**

FPG: fasting plasma glucose; HbA1c: glycated hemoglobin; HDL-c: high-density lipoprotein cholesterol; LDL-c: low-density lipoprotein cholesterol; eGFR: estimated glomerular filtration rate; ALP: alkaline phosphatase; P1NP: procollagen type 1 N-terminal propeptide; CTx: C-terminal telopeptide; iPTH: intact parathyroid hormone. Data are expressed as mean ± standard deviation for variables with a normal distribution and as median with interquartile range for variables with a non-normal distribution. Overall comparisons across FRAIL categories were performed using one-way ANOVA for normally distributed variables and the Kruskal–Wallis test for non-normally distributed variables. When appropriate, pairwise comparisons were performed using Bonferroni-adjusted post hoc tests when homogeneity of variances was met or Tamhane’s T2 test when variances were unequal. ^b^ Robust vs. Frail; *p* < 0.05; ^c^ Pre-frail vs. Frail; *p* < 0.05. Significant values in bold.

## Data Availability

The data presented in this study are available on request from the corresponding author due to privacy and ethical restrictions related to participant data.
